# Mammalian Lysine Histone Demethylase KDM2A Regulates E2F1-Mediated Gene Transcription in Breast Cancer Cells

**DOI:** 10.1371/journal.pone.0100888

**Published:** 2014-07-16

**Authors:** Wasia Rizwani, Courtney Schaal, Sateesh Kunigal, Domenico Coppola, Srikumar Chellappan

**Affiliations:** 1 Department of Tumor Biology, H. Lee Moffitt Cancer Center and Research Institute, Tampa, Florida, United States of America; 2 Department of Anatomic Pathology, H. Lee Moffitt Cancer Center and Research Institute, Tampa, Florida, United States of America; Peking University Health Science Center, China

## Abstract

It is established that histone modifications like acetylation, methylation, phosphorylation and ubiquitination affect chromatin structure and modulate gene expression. Lysine methylation/demethylation on Histone H3 and H4 is known to affect transcription and is mediated by histone methyl transferases and histone demethylases. KDM2A/JHDM1A/FBXL11 is a JmjC-containing histone demethylase that targets mono- and dimethylated Lys36 residues of Histone H3; its function in breast cancer is not fully understood. Here we show that KDM2A is strongly expressed in myoepithelial cells (MEPC) in breast cancer tissues by immunohistochemistry. Ductal cells from ductal carcinoma in situ (DCIS) and infiltrating ductal carcinoma (IDC) show positive staining for KDM2A, the expression decreases with disease progression to metastasis. Since breast MEPCs have tumor-suppressive and anti-angiogenic properties, we hypothesized that KDM2A could be contributing to some of these functions. Silencing KDM2A with small interfering RNAs demonstrated increased invasion and migration of breast cancer cells by suppressing a subset of matrix metalloproteinases (MMP-2, -9, -14 and -15), as seen by real-time PCR. HUVEC cells showed increased angiogenic tubule formation ability in the absence of KDM2A, with a concomitant increase in the expression of VEGF receptors, FLT-1 and KDR. KDM2A physically bound to both Rb and E2F1 in a cell cycle dependent manner and repressed E2F1 transcriptional activity. Chromatin immunoprecipitation (ChIP) assays revealed that KDM2A associates with E2F1-regulated proliferative promoters CDC25A and TS in early G-phase and dissociates in S-phase. Further, KDM2A could also be detected on MMP9, 14 and 15 promoters, as well as promoters of FLT1 and KDR. KDM2A could suppress E2F1-mediated induction of these promoters in transient transfection experiments. These results suggest a regulatory role for KDM2A in breast cancer cell invasion and migration, through the regulation of E2F1 function.

## Introduction

Methylation of the tail region of histones H3 and H4 has been correlated with transcriptional regulation and modulation of nucleosome function [1]. Histone methylation was long thought to be a stable and irreversible modification but the discovery of histone demethylases has led to the re-assessment of this concept [2], [3]. Two separate mechanisms of histone demethylation on lysine residues of histone H3 have been demonstrated - first, through amine oxidation by Lysine Specific Demethylase-1 (LSD-1/KDM1/BHC110/AOF2/KIAA0601) and second, through hydroxylation by JmjC-domain containing proteins [4]–[6]. Among the JmjC-domain containing histone demethylases discovered so far, KDM2A is known to selectively remove mono- and dimethyl groups only from histone H3K36 [7]. The activity of these demethylases is based on substrate conformity, and their cellular function on the proteins they physically interact with [8].

Non-methylated CpG islands constitute up to 70% of higher eukaryotic genes and KDM2A has been reported to functionally delineate the genomic architecture to differentiate the CpG islands from bulk chromatin by demethylating lysine 36 [9]. Further, while recent studies demonstrated that KDM2A maintains the heterochromatic state by binding to HP1 proteins and repressing transcription of centromeric satellite repeats [10], very little information is available on the precise function of KDM2A, especially in different cancers. Study on human prostate cancers showed low levels of KDM2A in prostate cancers [10] while a recent study states that high levels of KDM2A correlates with poor prognosis in NSCLC patients [11]. Here we studied the expression and function of KDM2A in human breast cancers and find that the expression of KDM2A is predominantly in the myoepithelial cells. Ductal cells of the breast tumors also show KDM2A expression but at considerably lower levels.

Histone modifying enzymes, including histone methyl transferases, histone acetyl transferases and histone deacetylases are known to modulate the proliferation of cells by regulating the activity of the E2F family of transcription factors. This is primarily through the retinoblastoma (Rb) tumor suppressor protein, which modulates E2F-mediated transcription by recruiting a variety of histone modifying proteins [12], [13], chromatin remodeling complexes [14], and DNA modifying enzymes [15]. It is well established that Rb mediated repression of the E2F transcription factors, especially E2Fs 1- 3, prevents cell cycle progression and the inactivation of Rb by phosphorylation mediated by cyclin dependent kinases facilitates S-phase entry [16]. Since certain histone demethylases are known to bind to Rb and regulate E2F function [17], [18] we examined whether KDM2A has similar functions, especially in breast cancer cells. Our results indicate that KDM2A represses migration and invasion of breast cancer cells, and inhibits angiogenic tubule formation by endothelial cells. KDM2A could bind to Rb and E2F1 and repress the transcriptional activity of E2F1. These results suggest that KDM2A might function in suppressing progression of breast cancer, by affecting cell invasion and angiogenesis.

## Materials and Methods

### Cell culture and siRNA transfections

T47D, MCF-7 and MDA-MB-231 cells (ATCC) were cultured in DMEM (Mediatech Inc.) containing 10% FBS. HUVECs (Lonza) were cultured in EGM-2 media (Lonza, Basel). MCF-7 cells were serum starved for 48 hr to render them quiescent and stimulated with serum for 2 hr, 4 hr, 6 hr, 8 hr and 18 hr for the western blots, co-immunoprecipitation experiments and ChIP assays and for 6 hr and 18 hr for immunofluorescence experiments.

For siRNA transfections, T47D, MCF-7 or HUVEC cells were transfected with non-targeting control siRNA or siRNA to KDM2A using oligofectamine reagent (Invitrogen). After 5 hr, the transfection media was replaced by DMEM containing 10% fetal bovine serum. 24 hr later, the cells were either serum starved and re-stimulated with serum or used directly depending on the experimental requirements. T47D and MCF-7 cells were transfected with 100 pmol of control siRNA and two different siRNAs to KDM2A, which were purchased from Ambion (KDM2Asi 1) and Santa Cruz Biotechnology (KDM2Asi 2). HUVEC cells were transfected with 75 pmol of control siRNA or KDM2Asi 2.

### Immunohistochemistry

The breast TMA (Breast TMA-2b, H. Lee Moffitt Cancer Center) incorporated 50 cores from normal breast tissue, 50 from DCIS, 50 from IDC without metastases, 50 from IDC with metastases and 50 lymph node metastases. The tissue array was stained for KDM2A using the standard protocol described previously [19]. In brief, the slide was incubated at 60°C for 1 hr, followed by two 10 minute incubations in xylene and was rehydrated in graded alcohol to water. Sodium citrate buffer (pH 6.0) was used for antigen retrieval. Primary antibody used was rabbit anti-human FBXL11 (KDM2A) at a 1∶800 dilution overnight at 4°C (KDM2A, Abcam). The rest of the staining was done following the manufacturer's protocol (VECTASTAIN Elite ABC kit (universal), Vector labs). For detection of bound antibody, DAB (3,3′-diaminobenzidine) peroxidase kit (Vector labs) was used. The tissue array was scanned on an Aperio Automatic Scanning System from Applied Imaging. Tumor sections were imaged at 200× magnification and analyzed by two pathologists (DC and AL). *p*-value was determined for statistical analysis by applying two-sided student's *t-*test and the data was considered significant when the *p*-value was less than .05. The experiment was conducted twice with two independent arrays.

### Invasion assays

The invasive ability of T47D and MCF7 cells transfected with a control siRNA or siRNA to KDM2A was assessed using a transwell Boyden chamber assay [20]. Following transfection, 20,000 T47D or MCF7 cells were plated in the top chamber in serum-free media and were allowed to migrate toward the bottom chamber containing DMEM with 20% FBS. The invading cells were quantitated after 18 h by counting six different fields at 200× magnification. Data presented is a mean of two independent experiments.

### Wound healing assays

A total of 100,000 T47D or MCF7 cells were grown to 70% confluency in a 6-well plate (Falcon Becton Dickinson). The cells were transfected with two independent siRNAs to KDM2A or a non-target control siRNA. After 5 hours or transfection, 10% DMEM was added to cells. They were then serum starved accordingly and three separate scratches were made per well after 24 h of transfection [20]. The cells were left in serum-free or complete media for 24 or 48 hrs depending on the cell line used and the wounds were imaged at 200× magnification using Evos FL Digital Fluorescence microscope. The data is representative of two independent experiments done in triplicate.

### Matrigel tube formation assay

A total of 100 µL of Matrigel (BD Biosciences) was added to 96-well plates followed by incubation for 60 minutes at 37°C. Control siRNA or KDM2A siRNA (2)-transfected HUVECs (12,000 cells/100 µL Matrigel) were seeded on the gels and incubated overnight at 37°C. Capillary tube formation was assessed using a Leica DMIL phase-contrast microscope [21]. The data is representative of two independent experiments done in triplicate.

### Real-Time PCR

Following transfections, MCF-7 cells were rendered quiescent by serum starvation and subsequently stimulated with serum. Unstimulated, serum-starved cells were used as control. Total RNA was isolated using RNeasy Mini Kit (Qiagen). First-strand cDNA was synthesized in a 20 µL reaction volume using the Bio-Rad iScript system (Bio-Rad Laboratories). Quantitative real-time PCR (qPCR) was performed with SYBR Green Supermix Taq Kit (Bio-Rad Laboratories) and analyzed on iCycler, MyiQ Single Color Real-Time PCR Detection System (Bio-Rad Laboratories), equipped with Optical System Software version 1.0. The PCR conditions were 10 minutes at 95°C, 1 minute at 55°C, 40 cycles of 15 seconds at 95°C followed by 30 seconds at 55°C. The real time PCR primers sequences for FLT-1 and KDR and 18S RNA were published previously [19], [22]. The mRNA expression data were normalized using 18S RNA as internal control, and the fold-change in the expression levels was determined using the quiescent cells as control. Each qPCR analysis was performed two independent times. Primer sequences are as follows: human KDM2A- Forward Primer 5′-CTCCCTTGAGCTTGGTTCTG-3′ and Reverse Primer 5′-AATCCACTTGGGTAGCAACG-3′ (product size-189 bp).

### Immunofluorescence and confocal microscopy

MCF-7 cells were rendered quiescent by serum starvation and subsequently stimulated with serum for 6 h and 18 h. The cells were stained as per the protocol described previously [23]. Double immunofluorescence experiments were conducted using rabbit anti-human KDM2A (1∶200 dilution) and mouse anti-human E2F1 (1∶200 dilution) antibodies. Secondary antibodies were goat anti-rabbit Alexa Fluor 555 (1∶1000 dilution) and goat anti-mouse- Alexa Flour 488 (1∶200 dilution). The cells were mounted with VECTASHIELD mounting medium with 4′ 6-diamidino-2- phenylindole (Vector Laboratories). Cells were observed using a Leica TCS SP5 confocal microscope (Leica Microsystems) at 630× magnification.

### GST-Binding Assays

GST, GST-Rb, GST-E2F1 were purified from bacterial cultures and bound to glutathione-Sepharose beads as previously described [23]. Beads were washed three times with PBS, and protein integrity was checked by polyacrylamide gel electrophoresis and Coomassie Blue staining. ^35^S-Methionine-labeled KDM2A was made using the rabbit reticulocyte lysate translation system according to the manufacturer's directions (Promega). 10 µl of labeled lysates was incubated with an equivalent amount of GST or GST-E2F1 or GST-Rb beads in protein-binding buffer [19]. Samples were incubated for 2 hr at 4°C and then washed in binding buffer six times. Bound proteins were eluted in gel loading buffer, resolved by SDS-PAGE gel electrophoresis and bands were visualized by autoradiography. The protein amount in control input lanes were approximately one-fifth of the total used in the binding assay.

### Lysate Preparation, immunoprecipitation and western blotting

Lysates from MCF7 cells stimulated with serum for different durations (0 h, 2 h, 4 h, 6 h, 8 h and 18 h) were prepared by Nonidet P-40 (Igepal-630) lysis as described earlier [19]. Lysates were subjected to SDS-PAGE and western blotted for KDM2A, Rb, E2F1 and actin. Physical interaction between proteins from 0 hr to 8 hr *in vivo* was analyzed by IP-Western blotting (as per the protocol by [24]) using 200 µg of total cell lysate for immunoprecipitation with 2 µg of the rabbit polyclonal KDM2A antibody (Abcam) or anti-rabbit IgG (irrelevant antibody) followed by western blotting with Rb and E2F1 antibodies. The results are representative of three independent experiments.

### ChIP assays

MCF-7 cells were serum starved and re-stimulated with serum for 2 h, 4 h, 6 h, 8 h or 18 h and chromatin immunoprecipitation (ChIP) lysates were prepared [19], [25]. Immunoprecipitation was conducted using antibodies against KDM2A and the association with specific promoters involved in E2F1-mediated proliferation was detected by PCR. Goat anti-rabbit secondary antibody was used as the control for all reactions. The sequences of the PCR primers were as follows: CDC25A promoter forward primer, 5′-TCT GCT GGG AGT TTT CAT TGA CCT C - 3′ and reverse primer, 5′-TTG GCG CCA AAC GGA ATC CAC CAA TC -3′; TS promoter forward primer, 5′-TGG CGC ACG CTC TCT AGA GC-3′ and reverse primer, 5′-GAC GGA GGC AGG CCA AGT G-3′.

Asynchronous MCF7 cell lysates were additionally immunoprecipitated with 5 µg of KDM2A, E2F1, or a non-specific target. Association with the promoters of MMP9, MMP14, MMP15, KDR, and FLT-1 were assessed by PCR. The sequences of the PCR primers used are as follows:

MMP9 forward 5′-TACGGTGCTTGACACAGTAAATCT-3′

MMP9 reverse 5′-AGGGCCTACTATGTGCCAGG-3′

MMP14 forward 5′-CCATAGGACTAGCCCAACTATGAG-3′

MMP14 reverse 5′-GAAGACTGACACCAGATGCTTGC-3′

MMP15 forward 5′-GGCTGCAGTTCACACATCTCA-3′

MMP15 reverse 5′-CTGTGACCAGATCTTGAAGCT-3′

FLT-1 and KDR primer sequences were obtained from [22].

### Cloning of E2.Luc reporter

E2.Luc vector was generated by subcloning sequence containing E2 sites from E2CAT vector into pGL3 basic vector. For this purpose, KpnI and NheI restriction enzyme sites were introduced by PCR using primers containing the restriction sites for KpnI and NheI at the 5′ and 3′-end respectively. The following primers were used to amplify the fragment to be cloned by PCR. Forward primer with KpnI site: 5′ GCAGGTACCGCATCGGTCGAAATTCTCAT-3′ and reverse primer with NheI site: 5′ ACGCTAGCCTCCTGAAAATCTCGCCAAG-3′. The PCR fragment and the pGL3 vector were then digested with KpnI and NheI restriction enzymes (New England Biolabs). These were run on 1.5% agarose gels and the bands were gel purified using Qiagen kit then ligated into the pGL3 basic vector. The cloned plasmid was then transformed into TOP10 cells and at least two positive clones were sequenced for the presence of E2 sequences and proper orientation.

### Transient transfections

MCF7 cells were transiently transfected with 0.5 µg MMP2-Luc, MMP9-Luc, MMP14-Luc and MMP15-Luc; and MDA-MB-231 cells with KDR-luc and FLT-1-luc using fuGENE HD (Roche Diagnostics) according to the manufacturer's protocol. Cells were co-transfected with 1 µg E2F1 and varying concentrations of KDM2A. 0.5 µg of pRL construct containing *Renilla reniformis* luciferase gene was used as normalizing control. Total DNA per well was adjusted to an equal level by adding the empty vector pGL3 or salmon sperm DNA. Luciferase assays were done by using Dual Luciferase Assay System (Promega). Relative luciferase activity was defined as the mean value of the firefly luciferase/Renilla luciferase ratios obtained from three independent experiments [24].

### Statistical analysis

All data have been graphically represented and statistically analyzed using Microsoft Office Excel 2007 (Microsoft Corporation). In all analyses, means were estimated and significance was calculated using a two-sided Student's *t-*test.

## Results

### KDM2A expression is elevated in breast myoepithelial cells

**Figure 1:**
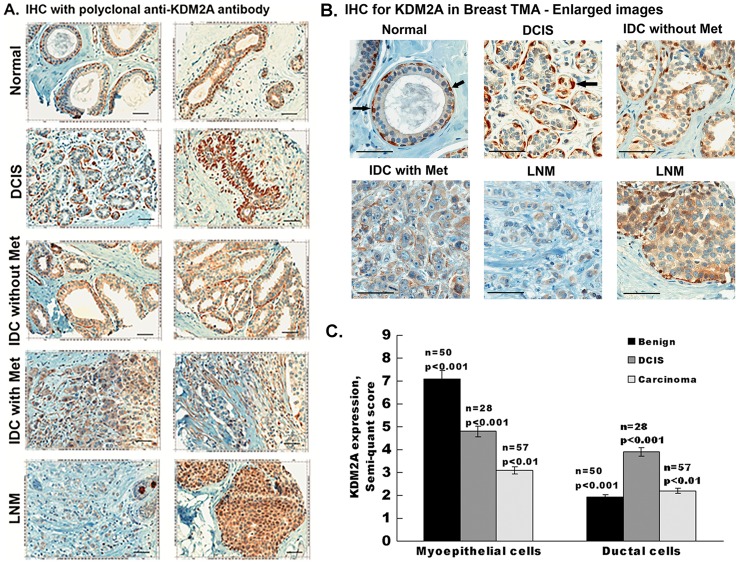
Myoepithelial cells of the breast express KDM2A. (A) Immunohistochemical staining of KDM2A on human breast cancer tissue microarray. Immunostaining was performed using rabbit anti-human KDM2A antibody and representative images of KDM2A expression in normal breast, Ductal Carcinoma in situ, Infiltrating Ductal Carcinoma with and without metastasis, lymph node metastasis are shown. Magnification is 200X, scale bar  = 50 µm. (B) Enlarged images of normal and cancer breast tissue sections. Arrows indicate positively stained myoepithelial cells. Scale bar  = 50 µm. (C) Quantitative analysis of KDM2A in breast tissue microarray. The Immunostaining of KDM2A was quantified by using semi quantitative scoring method based on cellularity and intensity of expression. The means of two independent arrays are shown. All *p*-values were calculated using a two-sided Student *t-*test.

We first analyzed KDM2A expression in breast cancer tissue microarray (TMA). The TMA had 50 normal, 50 DCIS (ductal carcinoma in situ), 50 IDC (Infiltrating Ductal Carcinoma) without metastasis, 50 IDC with metastasis and 50 LNM (Lymph Node Metastasis) cores. Of these, 50 were pathologically classified as normal/benign, 28 as DCIS and 57 as carcinoma; the levels of KDM2A were analyzed by immunohistochemistry. The semi-quantitative score was obtained by taking into consideration both cellularity and intensity of expression (Total Score  =  Cellularity x Intensity; a score of 3 represents >66% cellularity, 2 = 34%–65% and 1 = <33%. Intensity was scored as follows- 3 =  strong, 2 =  Moderate and 1 =  weak). The human breast consists of a branching ductal network with a surrounding outer layer of myoepithelial cells and an inner layer of polarized luminal epithelial cells [26]. Interestingly, it was found that myoepithelial cells (MEPC) of the breast showed the strongest staining for KDM2A. Benign (n = 50, p<0.001) and non-invasive ductal carcinoma in situ (n = 28, p<0.001) with intact myoepithelial layer stained strongly positive for KDM2A. Metastatic IDC had a few positive but very scarce myoepithelial cells, and most LNM (80±5%) were negative for KDM2A. Ductal cells from benign and carcinoma tissues stained weakly positive (p<0.001 and p<0.01) whereas ductal cells from DCIS showed moderate positivity for KDM2A (p<0.001). Figure 1A and 1B show representative enlarged images from different stages of breast cancer and Figure 1C is a graphical interpretation of the expression of KDM2A. Primary breast carcinomas are known to have increased number of luminal cells compared to myoepithelial cells [27] and many invasive breast carcinomas almost entirely lack myoepithelial cells. The differential expression of KDM2A raises the possibility that it might serve as a good marker for myoepithelial cells and therefore act as a biomarker for breast cancer progression from non-invasive to more invasive phenotype.

### KDM2A suppresses breast cancer cell invasion and migration

**Figure 2:**
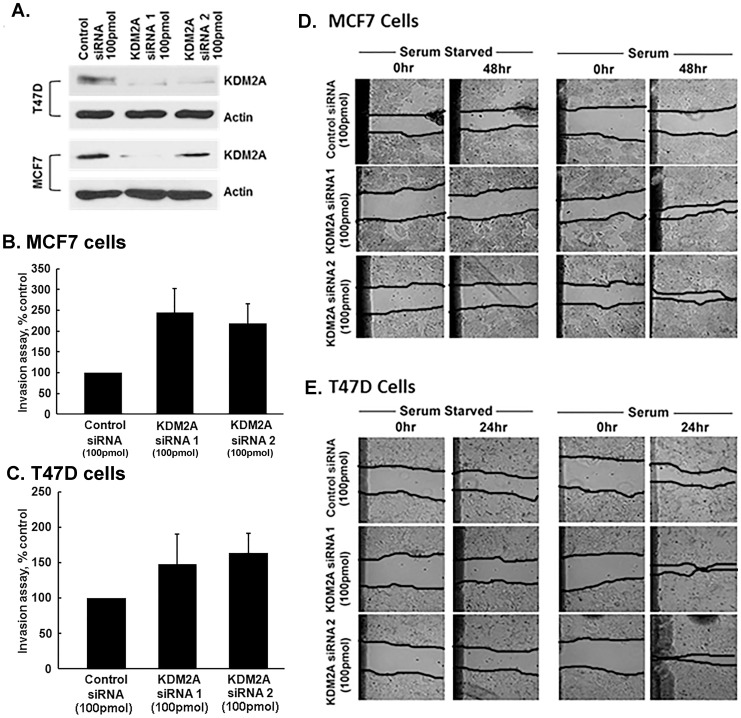
KDM2A suppresses invasion and migration of breast cancer cells. (A) Western blot analysis showing decreased KDM2A levels in T47D and MCF7 cells with two different siRNAs to KDM2A (Ambion and Santa Cruz) compared to control siRNA. (B) Silencing KDM2A by KDM2A siRNA 1 increased invasion in MCF7 cells by 145±23% (p<0.05) and KDM2A siRNA 2 increased invasion by 118±21% (p<0.05) when compared to control siRNA. (C) In T47D cells, KDM2A siRNA 1 enhanced invasion by 48±28% (p<0.1) and KDM2A siRNA 2 by 64±16% (p<0.05) when compared to control siRNA. (D) Both KDM2A siRNA1 (100 pmol) and KDM2A siRNA2 (100 pmol) transfected MCF7 cells migrated into the wound in the presence of serum at 48 hr when compared to control siRNA transfected cells. Serum starved cells did not migrate into the wound with or without KDM2A suppression. (E) T47D cells additionally show significant migration into the wound after transfection with KDM2A siRNA1 and KDM2A siRNA 2 in the presence of serum at 24 hr when compared to control siRNA. Serum starved cells did not migrate into the wound with or without KDM2A suppression. Magnification-200X.

MEPCs are required to maintain the normalcy of the breast epithelial architecture and have a tumor suppressor function [27]. Expression of KDM2A in MEPCs raised the possibility of a similar role for KDM2A in breast cancer cells, hence we examined the role of KDM2A in various cellular processes. As shown in Figure 2A, transfection of two independent KDM2A siRNAs, KDM2A siRNA 1 and KDM2A siRNA 2, at 100 pmol concentration reduced the protein levels significantly compared to control siRNA transfected cells, as observed by Western blot analysis. Experiments were conducted to investigate the role of KDM2A in the invasion of T47D and MCF7 breast cancer cells by Boyden chamber assay, using established protocols [19]. Cells were transfected with 100 pmol each of both KDM2A siRNAs 1 and 2, or a control siRNA and plated on the upper chamber of the transwell filters. The bottom chamber had DMEM with 20% FBS acting as a chemoattractant, the cells that migrated to the other side of the filter were stained with hematoxylin and counted. MCF7 cells showed a significant increase in invasion by about 145±23% with KDM2A siRNA 1 and 118±21% with KDM2A siRNA 2 while T47D cells showed a 48±28% increase in invasion with KDM2A siRNA 1 and a 64±16% increase in invasion with KDM2A siRNA 2, demonstrating that KDM2A inhibits the invasion of breast cancer cells (Figure 2B and 2C).

Experiments were performed to assess whether KDM2A affects the ability of cells to migrate on plastic. *In vitro* wound healing assays were carried out using T47D and MCF7 cells transfected with a control siRNA or two independent siRNAs specific to KDM2A. The cells were grown to confluency after transfection, were serum starved for 24 h, and were scratched with a pipet tip to generate an area devoid of cells. The cells did not migrate into the wound in serum-starved conditions; however, cells with abrogated KDM2A levels showed a marked increase in the migratory ability after addition of serum for 24 hr or 48 hr compared to control siRNA transfected cells (Figure 2D and 2E). Serum starved cells did not migrate into the wound at the same time points. Taken together, these results show that KDM2A represses invasion and migration of breast cancer cells, and depletion of KDM2A enhances these processes.

### KDM2A has anti-angiogenic effects by repressing VEGF receptors, FLT-1 and KDR

**Figure 3:**
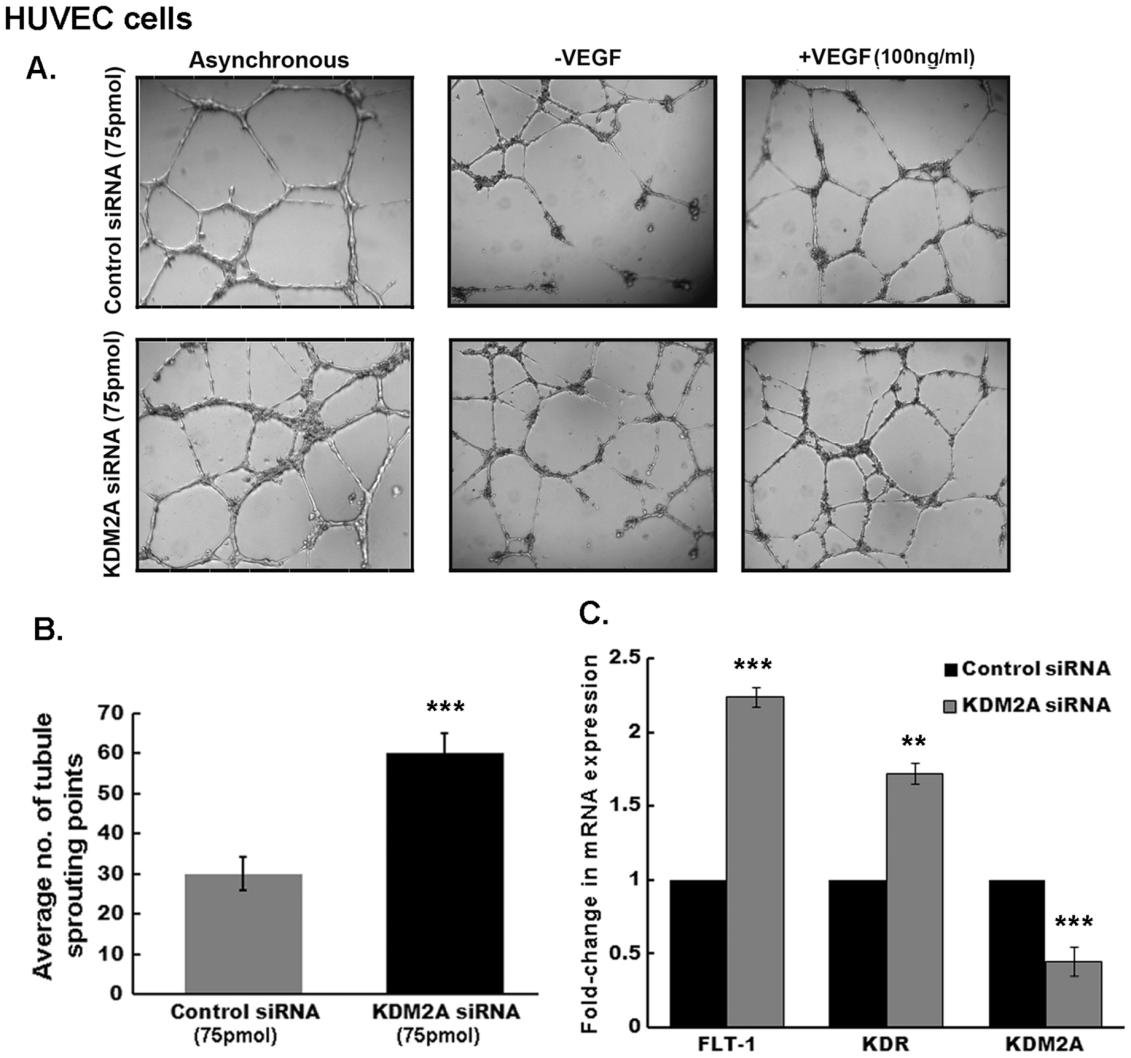
Silencing KDM2A enhances angiogenic tubulogenesis. (A) HUVEC cells were transfected with either control siRNA or KDM2A siRNA (75 pmol). 24 hr later, cells were plated on matrigel in complete media (asynchronous) or with or without 100 ng/ml VEGF. Images were captured 18 hr later using Leica inverted microscope and representative images are shown. (B) Tubule sprouting points were estimated using Image Pro software. Ablation of KDM2A showed significant increase (2-fold, p<0.01) in the number of sprouting points. (C) Real-Time showing increased mRNA expression of FLT-1 (2.2±0.11-fold, p<0.01) and KDR (1.7±0.9-fold, p<0.05) receptors upon silencing KDM2A expression. Simultaneous decrease in KDM2A mRNA levels (p<0.01) is seen with 75 pmol of KDM2A siRNA compared to control siRNA.

VEGF receptors, FLT-1 and KDR, are primarily expressed on endothelial cells. In addition, they are expressed on a variety of other cell types and tumors [28] including normal ductal epithelial cells, myoepithelial cells and ductal cells from tumors of the breast. MEPCs have been reported to be anti-angiogenic in function [29], therefore, we hypothesized that KDM2A could play a role in modulating angiogenesis. To determine the effect of KDM2A on angiogenic tubule formation in matrigel, we depleted KDM2A (using 75 pmol siRNA 1) in HUVEC cells and performed a matrigel tubulogenesis assay. Silencing KDM2A led to a significant increase (upto 2 fold) in the number of tubules and sprouting points (Figures 3A and 3B) compared to control siRNA transfected cells, both in the presence or absence of VEGF. Since FLT-1 and KDR are known to be regulated by E2F1 [22], we examined whether depletion of KDM2A could affect the expression of FLT-1 and KDR. RT-PCR results show that both FLT-1 and KDR mRNA expression increased in HUVECs depleted of KDM2A by 2.2±0.11-fold and 1.7±0.9-fold respectively (Figure 3C). Silencing of KDM2A was confirmed by RT-PCR for KDM2A (Figure 3C). These results suggest that KDM2A can regulate the angiogenic process, probably by controlling the E2F1-mediated expression of VEGF receptors.

### E2F1 interacts with KDM2A in a cell cycle dependent manner

**Figure 4:**
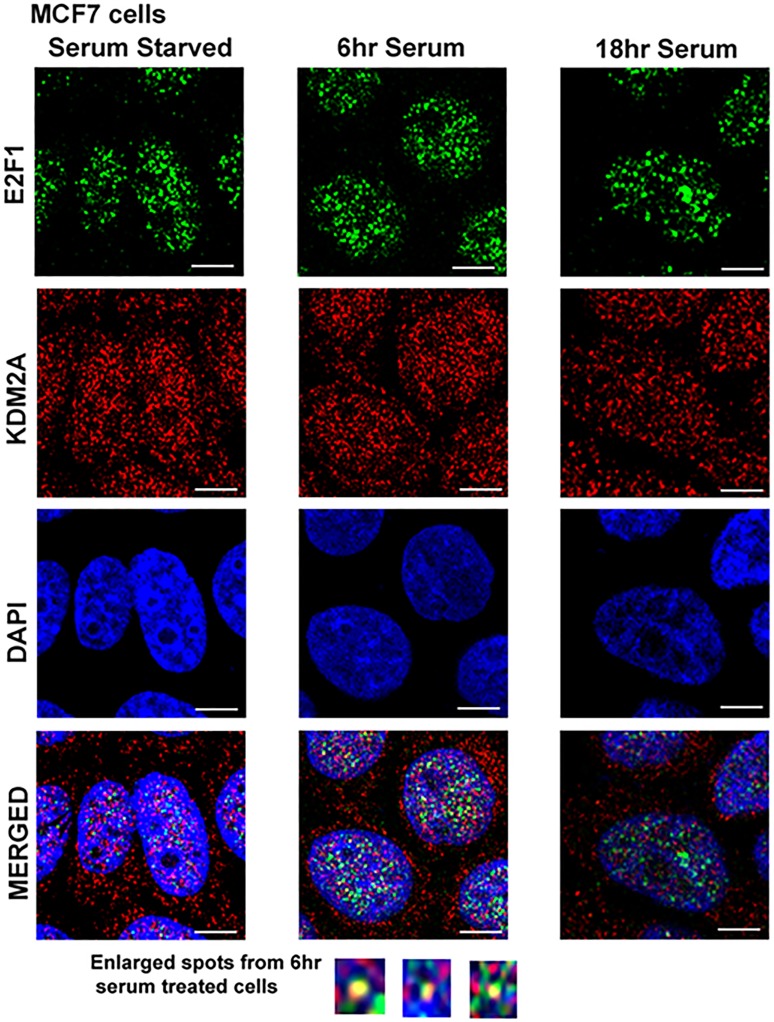
KDM2A co-localizes with E2F1 at 6 hr of serum stimulation. Quiescent MCF-7 cells were serum starved for 48 hr and serum stimulated for 6 hr and 18 hr. Cells were fixed, permeabilized for 5 min with 0.2% Triton X-100/PBS and immunostained for E2F1 (anti-mouse IgG, green) and KDM2A (anti-rabbit IgG, red). Cells were visualized by confocal microscopy. E2F1 was predominantly localized in the nucleus (upper panels), while KDM2A was more ubiquitously distributed in the cells (middle panels). Co-localization of E2F1 and KDM2A was observed in the nucleus at 6 hr of serum stimulation and disappeared totally by 18 hr (right panels). Images were captured at 630X oil using DM16000 inverted Leica TCS SP5 tandem scanning confocal microscope. Scale bar  = 200 µm. Pearson's correlation for co-localization at 6 hr was 1.0.

Previous studies have shown that VEGF receptors and certain MMPs are E2F1 regulated [22], [30]. Current data suggests that KDM2A works in conjunction with E2F1 to affect FLT-1 and KDR expression in endothelial cells and MMP expression in breast cancer cells. Further, many vital genes involved in cell proliferation and proliferation are regulated by E2F1 [19]. The finding that KDM2A could modulate many processes known to be regulated by E2F1 raises the possibility that KDM2A could be executing these functions through the modulation of the Rb-E2F1 transcriptional regulatory pathway. To test whether KDM2A affects Rb-E2F1 pathways, we first examined whether KDM2A interacts with E2F1 in MCF7 cells by a double immunofluorescence experiment. Quiescent (serum-starved) MCF7 cells were stimulated with serum for 6 hr and 18 hr and co-localization of KDM2A and E2F1 was assessed by a double immunofluorescence experiment followed by confocal microscopy [23]. E2F1 was expressed in the nucleus, while KDM2A was expressed predominantly in the nucleus but showed cytoplasmic staining as well at all time points studied. DAPI was used to stain the nuclei. Merged images showed a certain amount of co-localization of KDM2A with E2F1 in quiescent cells. At the same time, there was a strong co-localization 6 hr after serum stimulation and the co-localization of KDM2A with E2F1 was significantly reduced within 18 hr of serum stimulation (Figure 4). This suggests that KDM2A exists in association with E2F1 and this interaction is regulated through the cell cycle.

### KDM2A binds to Rb and E2F1 *in vitro* and *in vivo* and represses the transcriptional activity of E2F1

**Figure 5:**
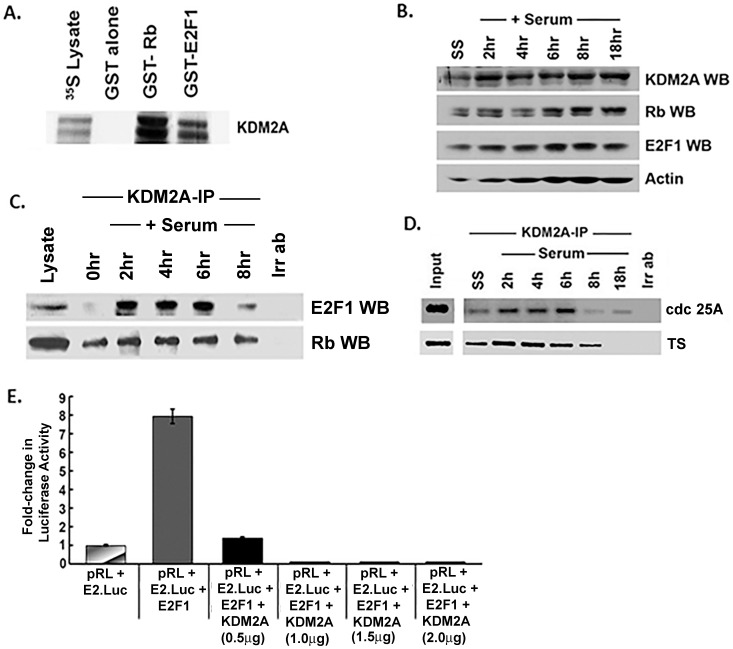
KDM2A interacts with Rb and E2F1 in a cell cycle specific manner. (A) GST pull down assay showing KDM2A binding to Rb and E2F1 *in vitro*. ^35^S-lysate lane has 1/10^th^ protein loaded. (B) MCF-7 cells were serum starved for 48 hr and serum stimulated for 2 hr, 4 hr, 6 hr, 8 hr and 18 hr. Western blotting showing protein expression of KDM2A, Rb, E2F1 and actin. There is no significant change in the expression of KDM2A and E2F1 at different time points when normalized to actin levels. Rb shows increasing hyperphosphorylation from 6 hr to 18 hr of serum stimulation. (C) KDM2A interacts with Rb and E2F1 *in vivo* in MCF-7 cells as seen by immunoprecipitation-western blot experiment. Interaction of KDM2A with E2F1 decreases at 8 hr of serum stimulation. (D) Chromatin Immunoprecipitation (ChIP) assays showing KDM2A occupancy on E2F1-regulated proliferative promoters, CDC25A and TS. KDM2A occupies the promoters at all time points from 0 hr to 6 hr, reduction is seen at 8 hr and complete absence of KDM2A from the promoters is observed at 18 hr of serum stimulation. (E) KDM2A significantly represses E2F1-mediated E2.Luc transcription in a dose-dependent manner in Renilla luciferase assay.

Based on the above data and the fact that Rb can bind to other histone modifying enzymes to repress E2F1 dependent transcription [12], [13], [15], we conducted additional experiments to confirm the interaction of KDM2A with Rb and E2F1. To assess whether KDM2A can bind directly to Rb or E2F1, *in vitro* GST binding assays were conducted using established protocols [23]. Essentially, ^35^S-labeled KDM2A was synthesized by *in vitro* transcription-translation in rabbit reticulocyte lysates and its binding to GST-Rb or GST-E2F1 immobilized on glutathione sepharose beads was assessed. It was found that KDM2A bound to GST-Rb and GST-E2F1 beads, but there was no binding to glutathione sepharose beads carrying uncoupled GST (Figure 5A). This suggests that KDM2A can bind to both Rb and E2F1 and this binding is probably direct. Immunoprecipitation-western blot experiments were conducted to assess whether the interaction observed in double immunofluorescence experiments as well as GST pull down assays could be replicated in a cell cycle dependent manner. Towards this purpose, MCF7 cells were rendered quiescent by serum starvation and stimulated with serum for 2 hr, 4 hr, 6 hr, 8 hr and 18 hr. Western blotting of the proteins of interest revealed that KDM2A and E2F1 expression was fairly consistent at different time points of serum stimulation, in relation to actin levels. Rb showed increased phosphorylation from 6 hr to 18 hr as seen by the increased intensity of the upper band consistent with prior studies. Actin was used as loading control (Figure 5B).

Lysates made from these cells were immunoprecipitated with a polyclonal antibody to KDM2A and the association of Rb and E2F1 assessed by western blotting. Immunoprecipitation with a secondary anti-Rabbit IgG was used as a negative control. The immunoprecipitation-western blotting experiments revealed that KDM2A was associated with Rb at all time points but its interaction was stronger at 2 hr, 4 hr and 6 hr of serum stimulation, whereas 8 hr showed a decrease in binding (Figure 5C). In contrast, E2F1 showed a robust binding to KDM2A at 2 hr, 4 hr and 6 hr of serum stimulation, and a sharp decrease in association at 8 hr of serum stimulation (Figure 5C), indicating that KDM2A may be regulating E2F1's transcription activity along with Rb. These experiments also suggest that KDM2A can bind to both Rb and E2F1, and these interactions might be differentially regulated.

Given that KDM2A could bind to E2F1 and Rb, chromatin immunoprecipitation (ChIP) assays were conducted to assess if KDM2A can be detected on E2F1-regulated proliferative promoters like CDC25A (cell cycle division homolog 25A) or TS (Thymidylate Synthase). ChIP assay lysates were prepared from quiescent MCF7 cells or those serum-stimulated for 2 hr, 4 hr, 6 hr, 8 hr and 18 hr using established protocols [21]. The lysates were immunoprecipitated with an anti-KDM2A antibody and the DNA obtained was amplified using primers for CDC25A and TS promoters. It was observed that KDM2A was associated with these promoters at 2 hr, 4 hr and 6 hr of serum stimulation; binding was reduced significantly at 8 hr and 18 hr of serum stimulation (Figure 5D). KDM2A could be detected on the promoters in quiescent cells as well. It appears that KDM2A is repressing the expression of these genes through E2F1. It is known that Rb is hyperphosphorylated and released from E2F1 by S-phase to facilitate proliferation of cells [31], therefore the absence of KDM2A on these promoters after 8 hr of serum stimulation indicates that KDM2A is repressing gene activation during G0 and G1 phase. The release of KDM2A from these promoters during S-phase could be facilitating E2F1's proliferative function. This data was substantiated with Renilla Luciferase transfection experiments.

Since KDM2A could bind to E2F1, transient transfection experiments were conducted in MCF7 breast cancer cells to assess whether KDM2A had an effect on the transcriptional activity of E2F1. MCF7 cells were transfected with an E2.Luc reporter. E2F1 (1 µg) was co-transfected alone or with different concentrations of KDM2A expression vector (0.5 µg, 1.0 µg, 1.5 µg or 2.0 µg). Transfection of E2F1 induced the expression of the E2.Luc reporter (8±0.5-fold increase) as expected; co-transfection of KDM2A significantly repressed E2F1-mediated transcription in a dose-dependent manner (Figure 5E). This demonstrates that KDM2A can suppress E2F1-mediated gene activation in transient transfection experiments.

### KDM2A represses MMPs and VEGF receptor expression by inhibiting the transcriptional activity of E2F1

**Figure 6:**
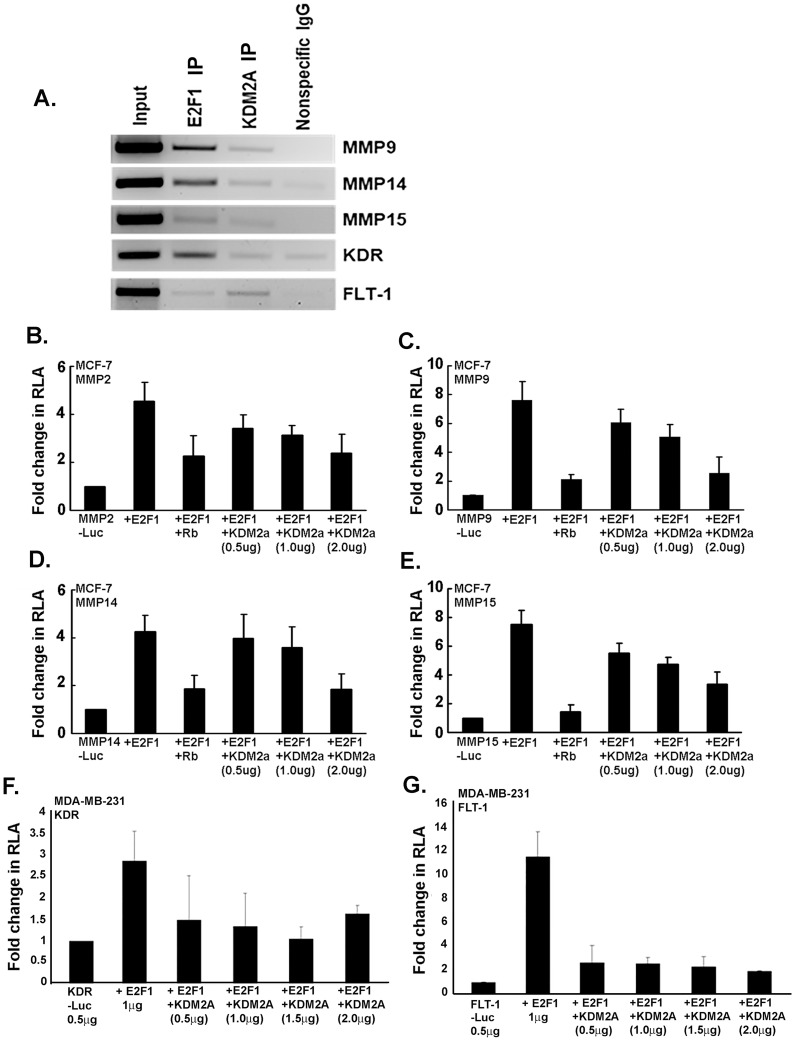
KDM2A represses transcriptional activity of MMPs and VEGF receptors by inhibiting E2F1-mediated transcription. (A) ChIP assay demonstrates occupancy of KDM2A on MMP9, -14, -15, KDR and FLT-1 similar to E2F1. (B, C, D, E) Transient transfection experiments in MCF-7 cells showed that E2F1 induces MMP2 (B), MMP-9 (C), MMP-14 (D) and MMP-15 (E) promoters, and this was repressed by co-transfection of KDM2A or Rb large pocket. (F, G) Transient transfection experiments in MDA-MB-231 cells demonstrate that KDM2A represses the transcriptional activity of E2F1 on KDR (F) and FLT-1 (G) promoters.

The above data lends support to the findings that KDM2A represses angiogenic and invasive properties in endothelial and breast cancer cells, probably through repressing E2F1-mediated transcription. We next examined the molecular mechanism underlying the KDM2A mediated repression of invasion and migration. Matrix metalloproteinases (MMPs) are known to play an important role in cancer cell invasion by mediating the degradation of extracellular matrix. Our earlier results have shown that multiple MMP genes, especially MM9, MMP15 and MMP15 are regulated by E2F1 transcription factor [30], and we examined whether KDM2A is associated with the promoters of these MMPs. Towards this purpose, ChIP lysates were prepared from asynchronously growing MCF7 cells and immunoprecipitated with E2F1 and KDM2A antibodies and their association with MMP9, MMP14 and MMP15 promoters examined by PCR using primers that span the E2F1 binding sites reported earlier [30]. As previously observed, E2F1 occupied the promoters of MMP9, MMP14 and MMP15; interestingly, we find that KDM2A is also associated with these promoters (Figure 6A), raising the possibility that KDM2A might repress MMP promoters by suppressing E2F1 activity. To examine whether KDM2A suppresses the MMP promoters, MCF7 cells were transiently transfected with luciferase reporter constructs driven by MMP2, MMP9, MMP14, and MMP15 promoters. It was found that co-transfection of E2F1 alone led to a significant induction of all the four promoters (Figures 6B, C, D and E); further, co-transfection of the large pocket region of Rb (Rb-LP) could repress the E2F1-mediated induction. Interestingly, co-transfection of KDM2A along with E2F1 considerably repressed the E2F1-mediated induction of the four MMP promoters in a dose-dependent manner. Taken together, these results indicate that the E2F1-mediated expression of MMP2, MMP9, MMP14, and MMP15 might be repressed by KDM2A, eventually hindering the migration and invasion of breast cancer cells.

Next we assessed the effect of KDM2A on the transcription of VEGF receptors, KDR and FLT-1. Results presented in Figure 3 demonstrate that KDM2A has anti-angiogenic effects and its silencing increased the expression of KDR and FLT-1 mRNA. ChIP assays revealed that similar to E2F1 [22], KDM2A also occupied the promoters of KDR and FLT-1 (Figure 6A). We further analyzed the repressive function of KDM2A on the VEGF receptor genes. MDA-MB-231 cells were transiently transfected with luciferase reporter constructs driven by KDR and FLT-1 promoters. It was found that co-transfection of E2F1 alone led to a significant induction of both KDR and FLT-1 promoters (Figures 6F and G); co-transfection of KDM2A along with E2F1 considerably repressed the E2F1-mediated induction of KDR and FLT-1. These results indicate that E2F1-mediated transcriptional activation of KDR and FLT-1 is significantly repressed by KDM2A, thereby inhibiting the angiogenic tubule formation.

## Discussion

KDM2A/FBXL11/JHDM1A is a histone-modifying enzyme that removes mono- and dimethyl groups from lysine residue 36 on histone H3. This particular histone modification is associated with transcriptional repression [7]. Recent studies have shown that KDM2A is required to sustain centromeric integrity and genomic stability, especially during mitosis [10]. Our findings reveal that KDM2A is expressed predominantly in the MEPCs of the human breast architecture. The myoepithelium lines the duct system in the breast [26] and any alterations in the myoepithelial cell layer reflect pathologic changes in the breast [26]. Primary breast carcinomas generally show a dramatic increase in luminal-to-myoepithelial cells, and many invasive breast carcinomas essentially lack MEPCs entirely [32]. KDM2A was present at low levels in the ductal cells. Normal ducts and intact ducts in the ductal carcinoma tissue sections showed myoepithelial cells strongly positive for KDM2A, suggesting a regulatory function of KDM2A in these cells. As the disease progressed towards malignancy, myoepithelial cells were found scattered in the breast cancer tissue sections. The possible mechanisms involved in the loss of MEPCs from around the duct for the invasion of tumor cells into the stroma need further investigation. However, in the recent studies, myoepithelial markers are frequently reported in breast cancer. Some metastatic lymph nodes were positive for KDM2A, suggesting that overexpression of KDM2A in breast might be an indication of metastasis. Lack of myoepithelial layer around the duct is an important diagnostic feature of ductal carcinoma in situ (DCIS) and also an indication of the disappearance of a major obstacle for stromal invasion [33]. Overexpression of KDM2A in lung cancer was associated with poor prognosis and is believed to promote lung tumorigenesis by repressing DUSP3 (dual-specificity phosphatase 3) and activating ERK1/2 [11]. Whether the same mechanism holds true for the metastatic breast tumors overexpressing KDM2A needs to be evaluated.

MEPCs have been considered as tumor suppressive cells in breast. A number of tumor suppressor proteins such as p63, p73, 14-3-3- sigma, maspin and Wilm's Tumor protein are preferentially expressed in MEPCs [6], [34]. The expression of KDM2A in the MEPCs also suggested a tumor suppressor function for KDM2A, consistent with the myoepithelial cell function in the breast; supporting this contention, KDM2A repressed migration and invasion of breast cancer cells. The data presented here suggests that KDM2A might be regulating the invasive and migratory properties through modulating the transcriptional activity of E2Fs. E2F family of transcription factors also bind to their target promoters and regulates expression of a variety of genes involved in cell proliferation, apoptosis and survival, in addition to invasion. The transcriptional regulatory functions of E2F are to a great extent affected by the presence of co-activators and co-repressors, many of which are recruited to the salient promoters through the Rb protein. For example, Rb recruits various transcriptional corepressors, including histone deacetylase 1 (HDAC1), DNA methyltransferase, and Polycomb proteins as well as chromatin-remodeling complexes like Brg and Brm [13], [14], [35]–[40]. Thus, it was likely that histone demethylases could also be a part of Rb-E2F1 complex interacting proteins. Supporting this contention, KDM1 has been shown to interact with Rb and regulate Rb-E2F1 interaction in Epstein-Barr virus [41].

Our results demonstrate that KDM2A associated with Rb and E2F1 in a cell cycle dependent manner in breast cancer cells indicating that this interaction could probably be regulating cell cycle progression. Both histone methyl transferases and demethylases are known to impact cell cycle by directly modulating expression of many cell cycle genes. In yeast, G1/S transition involves dimethylation and not trimethylation of H3K27 that serves as an epigenetic memory for genes transcribed in the previous cell cycle [42]. Similarly, in human T cells, genes active in G1 phase are marked with H3K4me3 during G0 [43]. KMT6 in mammalian cancer cells regulate genes that in turn control cell cycle progression such as Cyclin A2, D1 and E1 [44]. Another histone demethylase, KDM7B (PHF8) facilitates G1/S transition by regulating E2F1 target genes [45]. It is possible that KDM2A is also exerting similar functions in the cells, by altering the methylation status of histone H3 and H4. It is also possible that KDM2A might be having a direct effect on E2F1 protein; these aspects require additional investigation.

It is believed that MEPCs suppress stromal invasion of tumor cells by secreting anti-angiogenic and anti-invasive factors [46]. Not surprisingly, KDM2A depletion resulted in increased VEGF receptors, FLT1 and KDR activity. We have previously shown that E2F1 occupies the promoters of FLT1 and KDR and enhances the expression of these receptors [22]. Simultaneously, KDM2A suppressed different MMPs in the presence of E2F1. MMPs not only facilitate invasion of cancer cells by degrading extracellular matrices but also facilitate angiogenesis [47]. KDM2A could be negatively regulating MMP function by repressing E2F1 to maintain normal cell function and integrity. These data implicate that KDM2A might be regulating cellular biological processes like migration, invasion and angiogenesis by regulating E2F1 function. Whether KDM2A has an impact on other E2F family members needs to be determined.

**Figure 7:**
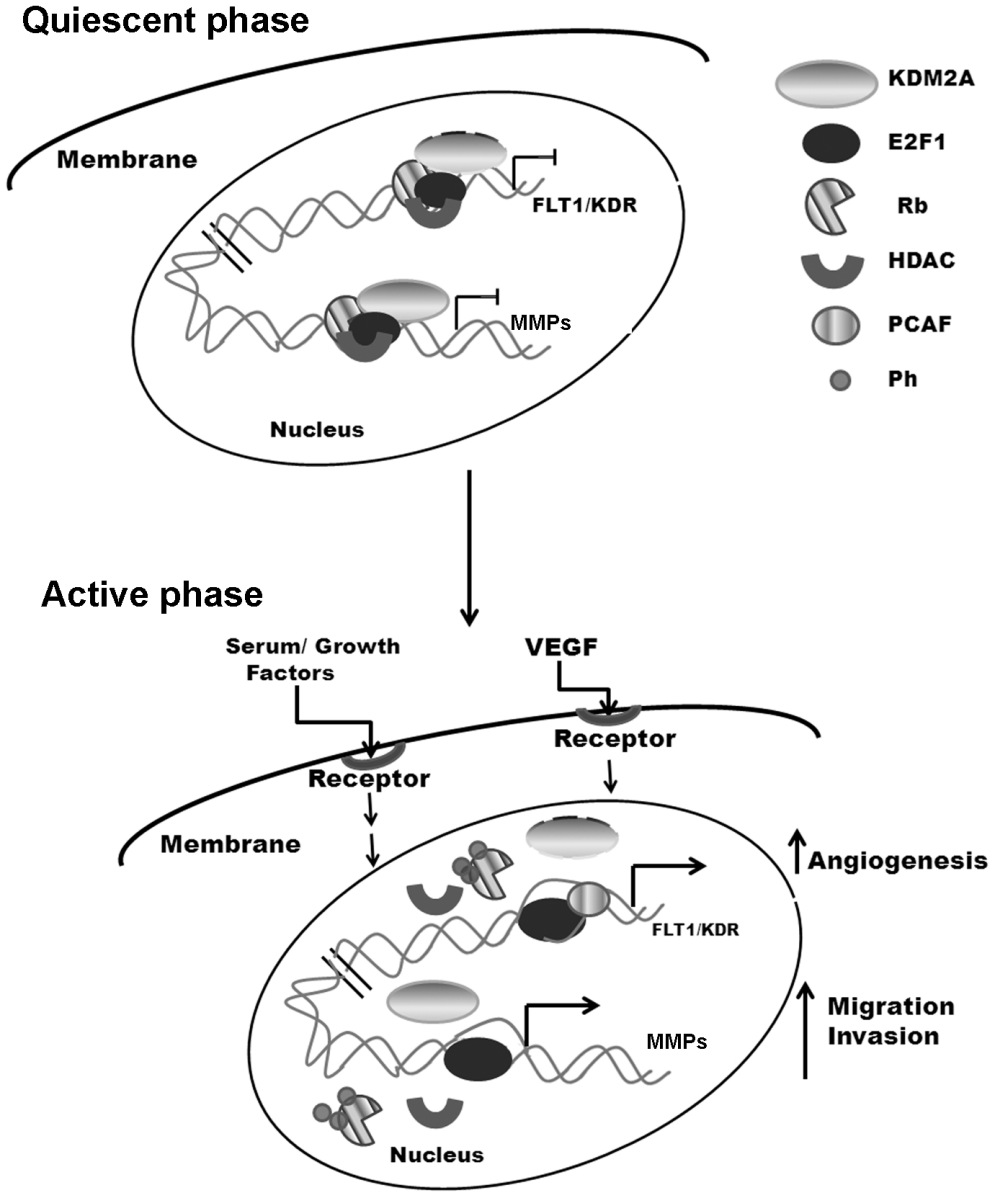
A model depicting KDM2A function in a mammalian cell. It can be implied that KDM2A regulates Rb-E2F1 function in the progression of the cell cycle. In a quiescent state, KDM2A represses E2F1 functions on various promoters. Addition of VEGF or serum dissociates KDM2A from these promoters facilitating various cellular processes by transcriptional activation leading to enhanced angiogenesis, invasion and migration of cells.

In conclusion, our observations suggest that KDM2A functions as a co-regulator of E2F1-mediated gene transcription, angiogenesis and metastasis (Figure 7). These findings also raise the possibility that KDM2A is recruited to the target genomic locations on chromatin at least in part through E2F1. Further, the fact that KDM2A is expressed in myoepithelial cells requires further investigation of KDM2A as a prognostic marker, in angiogenesis and metastasis of breast cancer.
